# Printed Transformable Liquid-Metal Metamaterials and Their Application in Biomedical Sensing

**DOI:** 10.3390/s21196329

**Published:** 2021-09-22

**Authors:** Yi Ren, Minghui Duan, Rui Guo, Jing Liu

**Affiliations:** 1 Department of Biomedical Engineering, Tsinghua University, Beijing 100084, China; ren-y18@mail.tsinghua.org.cn (Y.R.); dmh17@mails.tsinghua.edu.cn (M.D.); guor16@tsinghua.org.cn (R.G.); 2 Technical Institute of Physics and Chemistry, Chinese Academy of Sciences, Beijing 100190, China; 3 School of Future Technology, University of Chinese Academy of Sciences, Beijing 100049, China

**Keywords:** liquid metal, metamaterials, electromagnetic element, biomedical sensing

## Abstract

Metamaterial is becoming increasingly important owing to its unique physical properties and breakthrough applications. So far, most metamaterials that have been developed are made of rigid materials and structures, which may restrict their practical adaptation performances. Recently, with the further development of liquid metal, some efforts have explored metamaterials based on such tunable electronic inks. Liquid metal has high flexibility and good electrical conductivity, which provides more possibilities for transformable metamaterials. Here, we developed a new flexible liquid-metal metamaterial that is highly reconfigurable and could significantly extend the working limit facing current devices. The printed electronics method was adopted to fabricate artificial units and then construct various potential transformable metamaterials. Based on metamaterial theory and printing technology, typical structured flexible liquid-metal electromagnetic metamaterials were designed and fabricated. The electronic and magnetic characteristics of the liquid-metal-based electromagnetic metamaterials were evaluated through simulated analysis and experimental measurement. Particularly, the potential of liquid-metal metamaterials in biomedical sensing was investigated. Further, the future outlook of liquid-metal metamaterials and their application in diverse categories were prospected.

## 1. Introduction

Metamaterials, artificial composite materials with supernormal physical properties that cannot be found in nature, were first systematically proposed in 1968 [1]. A significant breakthrough was made in 2000 when the theoretical model thus evolved was designed [2]. Thereafter, experiments verified a negative index of refraction [3], and materials with both negative permittivity and permeability in microwave range were created [4,5,6]. Since then, investigations on metamaterials developed rather quickly [7] and more unique properties have continued to be discovered, including reversed the Cherenkov radiation effect [8,9], reversed Doppler effect, reversed Goos–Hänchen shift [10], evanescent-wave amplification and transmission [11], perfect lens [2], and so on. Generally, the abnormal electromagnetic performance of metamaterials is highly dependent on the artificial structure rather than the composition. Various metamaterials based on periodic structures have been put forward [12,13,14,15,16,17,18,19,20,21,22,23,24,25,26].

**Figure 1:**
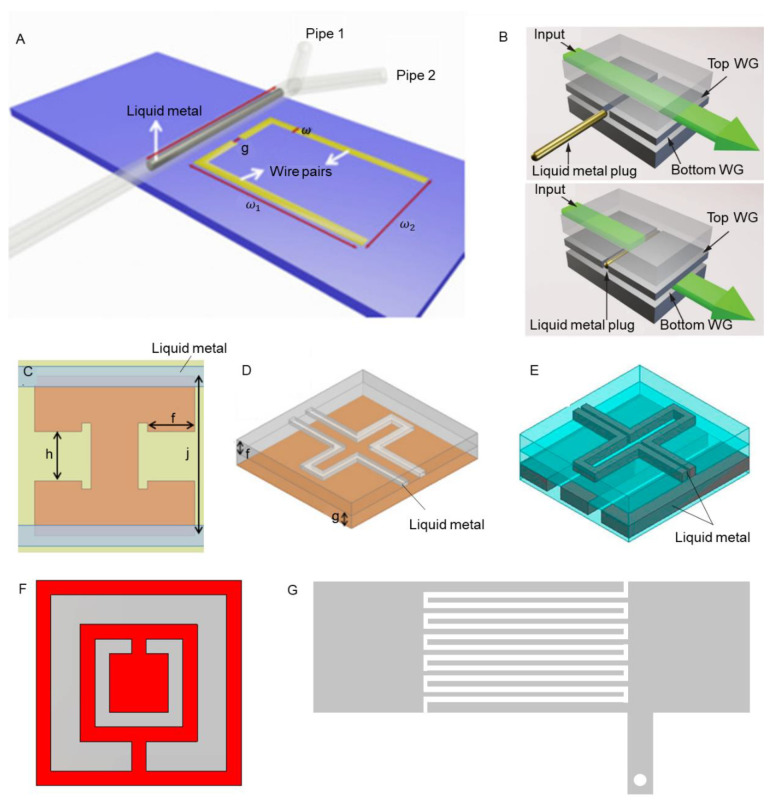
Illustration of liquid-metal metamaterials. (**A**) switchable EIT metamaterial; Reproduced with permission from ref. [36]; Copyright 2019 Optics Express. (**B**) electrically reconfigurable terahertz signal processing device with a liquid-metal plug; Reproduced with permission from ref. [37]; Copyright 2018 Nature Communications. [37] frequency-switchable metamaterial absorber; Reproduced with permission from ref. [38]; Copyright 2015 Sensors. (**D**,**E**) flexible metamaterial absorbers based on PDMS and liquid metal; Reproduced with permission from ref. [39]; Copyright 2015 Optics Express. Reproduced with permission from [40]; Copyright 2016 Sensors, respectively. (**E**,**F**) SRR structure; (**G**) transmission line configurations. (Reproduced with permission.)

Until now, most of the existing metamaterials are made of rigid materials and structures. When fabricated, their functions and capabilities are generally fixed, which have limited practical adaptation performances. To break through these limitations, attempts have been made to fabricate transformable metamaterials based on liquid metal due to its unique properties. As was gradually realized, liquid metal is a kind of metal that can maintain a liquid state at room temperature [27]. Except for toxic metals such as mercury, gallium, and its alloy own the properties of low toxicity, high fluidity, and excellent electrical and thermal conductivity, which can even be adopted as biomaterials [28,29,30,31,32]. Several types of metamaterials have been proposed and designed based on liquid metal as well. For instance, terahertz metamaterial absorbers fabricated by liquid metal were demonstrated, and their performance was highly dependent on the thermal properties of liquid metal [33,34]. Additionally, a metasurface with frequency-agile and wide-angle was realized by liquid metal recently [35]. Moreover, with the introduction of liquid metal, metamaterials have the advantages of being switchable, stretchable, and frequency tunable. Firstly, exploiting the fluidity of liquid metal, the properties of metamaterials can be switched on and off. As shown in Figure 1A, Xu et al., fabricated a switchable EIT metamaterial, which could be switched on or off by liquid metal [36]. The interference of a copper wire pair and liquid metal rod led to an electromagnetically induced transparency-like spectrum. Figure 1B presents an electrically reconfigurable terahertz signal processing device [37]. The liquid-metal plug could be repositioned by an external voltage and then change the electromagnetic coupling between the two waveguides. Ling et al., injected liquid metal into a microfluidic channel to form a frequency-switchable metamaterial absorber (Figure 1C) [38]. It has been proven that the resonant frequency was switched from 10.96 GHz to 10.61 GHz when liquid metal was present. Secondly, metamaterials based on liquid metal can maintain benign flexibility. Ling et al. and Kim et al. designed stretchable metamaterial by injecting liquid metal into polydimethylsiloxane (PDMS) microfluidic channels [39,40], as shown in Figure 1D,E. The absorption frequency was changed when being stretched, which could be used for wireless sensing and monitoring. Furthermore, microlattice metamaterials composed of liquid metal and polymer were fabricated, which had high fracture toughness and damage recoverability [41]. Finally, liquid metal has potential for realizing frequency-tunable metamaterials. Liquid metal is fluidic and electrically driven. Thus, metamaterials made by liquid metal could be reshaped by external force and electric field. Then, the working frequency of the metamaterials would be changed as well [37,42,43]. Obviously, liquid metal has shown great potential in metamaterials, which can be applied to soft robots, flexible sensors, and biomedical applications [41,44].

In this article, we attempted to systematically propose a new liquid-metal transformable metamaterial [43], which is reconfigurable in structure and easy to quickly print out. For illustration purposes, transformable Split-Ring Resonator (SRR) units thus made were investigated both theoretically and experimentally. The potential of liquid-metal-based metamaterials in soft sensing was further explored. Overall, compared with the classical rigid metamaterials, the liquid-metal transformable metamaterials are highly reconfigurable and may break the working limit of existing technologies. The functions of such devices thus made can be regulated by an electrical or magnetic field. And soft circuits designed by liquid metal can be twisted and stretched to supply various electromagnetic functions. To fabricate liquid-metal-based SRR units and construct such transformable metamaterials, liquid-metal printing technology was adopted, which is a recent technology that has been gradually applied to the fast fabrication of flexible circuits [44,45,46,47,48]. The liquid-metal-based SRR units were fabricated on paper and soft polymer substrate simultaneously. Moreover, typical principles for constructing and running such metamaterials were outlined. In addition, the electromagnetic function of liquid-metal-based SRR units was evaluated, demonstrating that liquid metal can be used for making transformable metamaterials. Compared with traditional rigid PCB SRRs, liquid-metal-enabled SRRs are more convenient to fabricate and use and provide an edge in changeable working characteristics. Moreover, liquid-metal-based transformable metamaterials are expected to play a significant role in future research and application, especially for biomedical sensing and detecting.

## 2. Simulation

**Figure 2:**
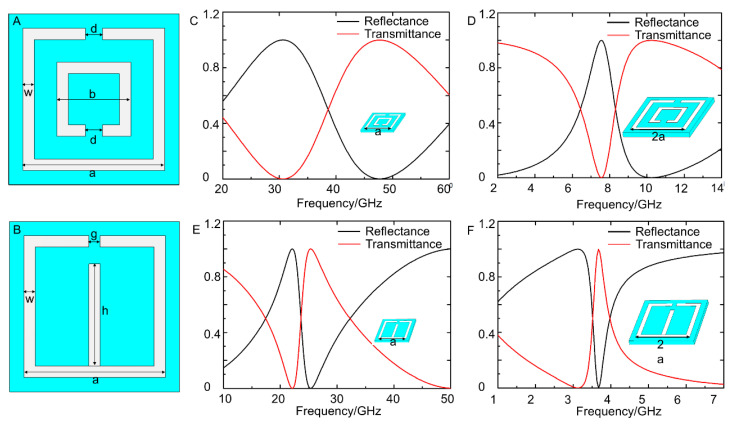
(**A**) The design of a DSRR unit; (**B**) the design of an ESRR unit; (**C**,**D**) the simulated reflectance–transmittance curves of DSRR units when the outer side length was 2.5 mm and 5 mm, respectively; (**E**,**F**) the simulated reflectance–transmittance curves of ESRR units at 2.5 mm and 5 mm, respectively.

To realize liquid-metal-based electromagnetic metamaterials, double Split-Ring Resonator (DSRR) and E-type Split-Ring Resonator (ESRR) units were designed. Figure 2A,B depict the specific structure of those SRR units. The parameters were designed as: a = 2.5 mm, b = 1.3 mm, d = 0.3 mm, w = 0.2 mm, g = 0.2 mm, h = 2 mm. Generally, SRR units can be seen as LC equivalent circuits, and the resonant frequency ($\omega_{0}$) of metamaterials highly depends on the structure and size of the SRR units. Herein, the electromagnetic properties of metamaterials constituted of different SRR units were explored through simulated analysis.

CST Microwave Studio was adopted to simulate the electromagnetic properties of DSRR-based metamaterials and ESRR-based metamaterials. From Figure 2C,D, it is concluded that the resonant frequency decreased as the size of the DSRR unit increased. When the outer side length was 2.5 mm, the transmittance of DSRR-based metamaterials dropped to the bottom at 30.8 GHz, which meant that its resonant frequency was 30.8 GHz. However, when the outer side length was 5 mm, the resonant frequency transferred to 7.6 GHz. Similar phenomena were observed from ESRR-based metamaterials, as shown in Figure 2E,F. Those results proved that the resonant frequency was influenced by the structure and size of the minimum repeating unit.

**Figure 3:**
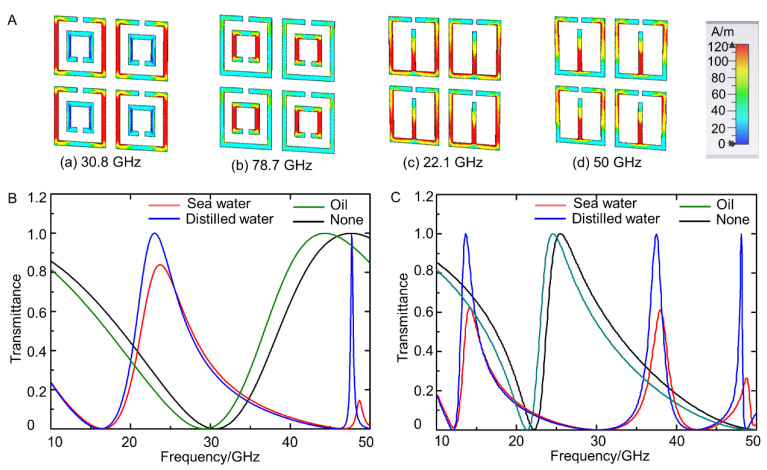
(**A**) Simulation of the surface current distribution: (**a**) surface current distribution of DSRR units at 30.8 GHz; (**b**) surface current distribution of DSRR units at 78.7 GHz; (**c**) surface current distribution of ESRR units at 22.1 GHz; (**d**) surface current distribution of ESRR units at 50 GHz. (**B**) The transmittance curves of DSRR units when coated by different matter; (**C**) transmittance curves of ESRR units when coated by different matter.

Figure 3A shows the surface current distribution of DSRR units and ESRR units at the resonant frequency when the outer side length was 2.5 mm. For DSRR units, when the frequency was 30.8 GHz, the resonance occurred due to the outer larger ring, while the resonance at 78.7 GHz was mainly attributed to the inner smaller ring. For ESRR units, the resonances occurred at the part away from or near the opening. The part away from the opening played a major role at 22.1 GHz, and near the opening at 50 GHz. Furthermore, the sensing ability of these two structures was explored. To monitor the change of transmittance, the surface of metamaterials was coated by sea water, distilled water, and oil. As shown in Figure 3B,C, oil and water could be well distinguished. For sea water and distilled water, the difference in resonant frequency was not obvious, but the values of transmittance at resonant frequency were not the same. Clearly, metamaterials coated by distilled water had higher transmittance at resonant frequency. These results primarily proved the potential application of liquid-metal metamaterials in sensing and monitoring in theory.

## 3. Experimental Verification

**Figure 4:**
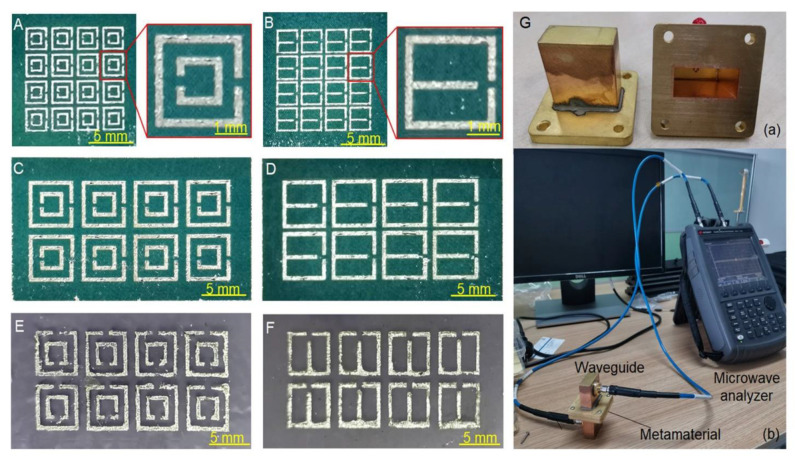
Fabrication and test of metamaterials based on liquid metal. (**A**) An array of 16 (4 × 4) DSRR units when the outer side length was 2.5 mm; (**B**) an array of 16 (4 × 4) ESRR units when the outer side length was 2.5 mm; (**C**) an array of 8 (2 × 4) DSRR units when the outer side length was 5 mm; (**D**) an array of 8 (2 × 4) ESRR units when the outer side length was 5 mm; (**E**) DSRR units on Ecoflex; (**F**) ESRR units on Ecoflex; (**G**) waveguide measurement setup: (**a**) a pair of waveguides; (**b**) assembly drawing.

Based on simulation results, metamaterials composed of DSRR units and ESRR units were fabricated successfully through printing technique, as shown in Figure 4A–D. Arrays based on DSRR units and ESRR units were printed on paper. The outer side length was designed as 2.5 mm and 5 mm, respectively. Moreover, metamaterials fabricated on paper can be further transferred onto elastic substrate, such as PDMS and Ecoflex. For instance, Figure 4E,F are photos of metamaterials transferred onto Ecoflex. Attributed to the fluidity of liquid metal, they had good flexibility, which could tolerate bending and stretching to a certain extent. Thus, liquid-metal metamaterials can be quickly obtained by printing technique and display the advantages of being transformable and flexible.

**Figure 5:**
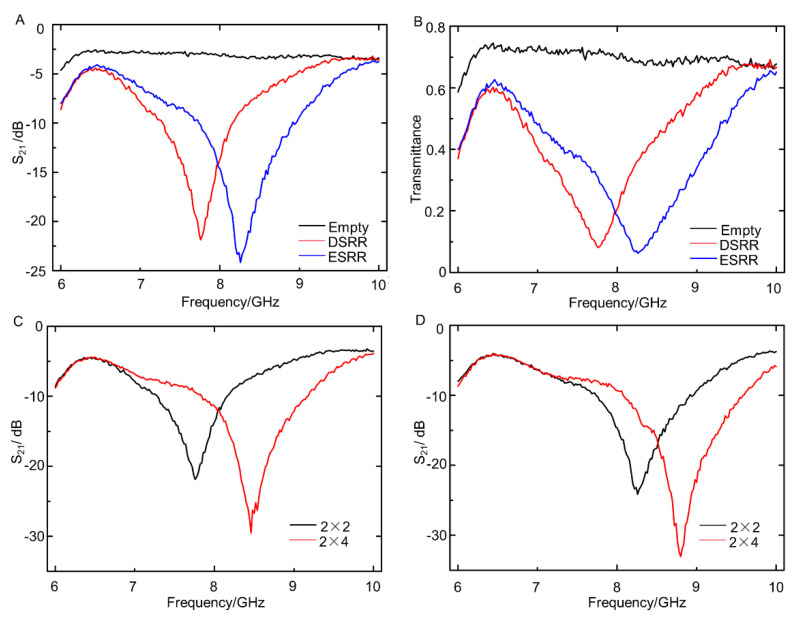
Electromagnetic properties of metamaterials based on SRR arrays: (**A**) the S_21_ parameter of metamaterials with different structures; (**B**) the transmittance curve of metamaterials with different structures; (**C**) the S_21_ parameter of DSRR-based metamaterials composed of different arrays; (**D**) the S_21_ parameter of ESRR-based metamaterials composed of different arrays.

To measure the electromagnetic properties of fabricated liquid-metal metamaterials, a waveguide and microwave analyzer were utilized, as shown in Figure 4G. The S_21_ parameter in the range of 6–10 GHz was measured, and the transmittance was calculated accordingly. As shown in Figure 5A, when the waveguide device was unloaded, the S_21_ parameter fluctuated around −3 dB, and there was no obvious difference when the frequency changed. When DSRR-based or ESRR-based metamaterials were present, the S_21_ parameter varied greatly with frequency. For DSRR-based metamaterials, the S_21_ parameter reached the minimum value when the microwave frequency was 7.76 GHz, indicating that the resonant frequency of DSRR-based metamaterials was 7.76 GHz. For ESRR-based metamaterials, the resonant frequency was 8.26 GHz. Figure 5B displays the transmittance curve calculated through the S_21_ parameter. The transmittance of DSRR-based and ESRR-based metamaterials was lower than 0.1 at resonant frequency, proving that the fabricated metamaterials had a unique electromagnetic response.

Meanwhile, metamaterials with different repeating units were investigated as well, including 2 × 2 arrays and 2 × 4 arrays. Figure 5C,D show the influence of repeating units on DSRR-based and ESRR-based metamaterials respectively. It could be concluded that when the number of the repeating units increased, the resonant frequency slightly shifted to the high-frequency region. Simultaneously, the resonance phenomenon was more intense, which meant that the transmittance of microwaves became lower at resonant frequency. Therefore, the electromagnetic properties of metamaterials based on SRR arrays could be further regulated by changing the number of repeating units.

**Figure 6:**
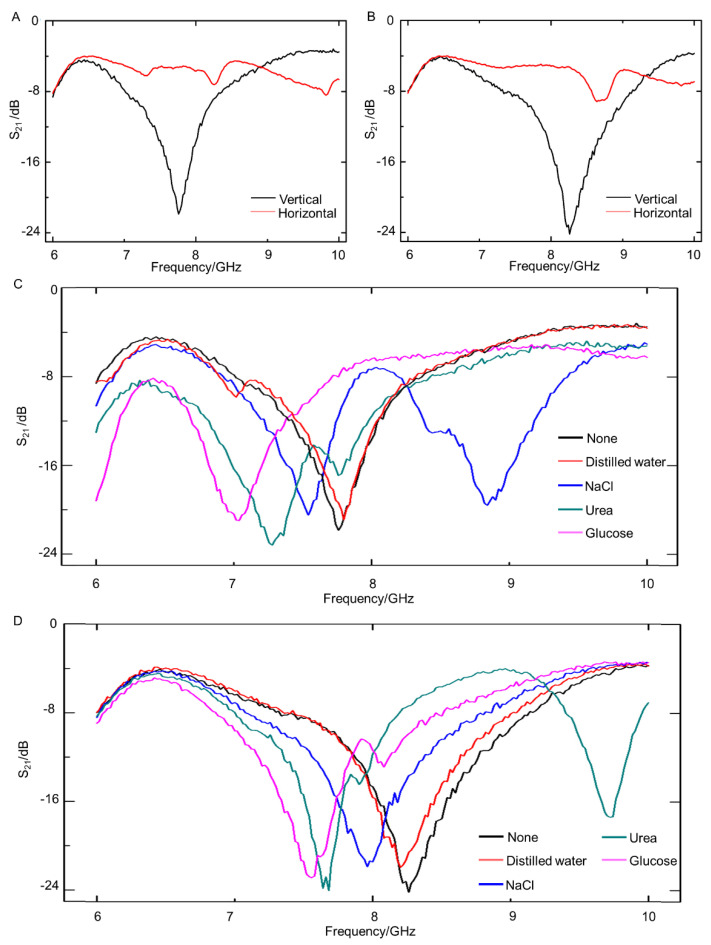
The sensing performance of liquid-metal metamaterials: (**A**) the S_21_ parameters of DSRR-based metamaterials in vertical and horizontal positions; (**B**) the S_21_ parameters of ESRR-based metamaterials in vertical and horizontal positions; (**C**) the S_21_ parameters of DSRR-based metamaterials when deposited by different substances; (D) the S_21_ parameters of ESRR-based metamaterials when deposited by different substances.

Furthermore, the sensing performance of liquid-metal metamaterials was tested, including direction monitoring and substance detecting. There were openings in DSRR and ESRR units, and the orientation of these openings could greatly influence the electromagnetic response of metamaterials. Herein, we arranged the fabricated metamaterials in vertical and horizontal positions to measure the S_21_ parameter. As shown in Figure 6A, the S_21_ parameters of DSRR-based metamaterials in vertical and horizontal positions were completely different. When DSRR-based metamaterials were arranged in a vertical position, the S_21_ parameters ranged between −4 dB and −24 dB and there was a distinct resonant peak. It could be concluded that the electromagnetic response in a vertical position was frequency dependent. However, when in a horizontal position, the S_21_ parameters just fluctuated between −4 dB and −8 dB, indicating that the microwave transmittance was at a high level and there was no obvious resonant peak. Similar phenomena on ESRR-based metamaterials can be observed in Figure 6B. Since there existed a significant difference between the electromagnetic response curves of the metamaterials in the vertical position and the horizontal position, the orientation of the openings of metamaterials can be judged by the trend of the curve. Thus, metamaterials could be used for direction monitoring.

Additionally, it was found that when different molecules were deposited on the surface of the metamaterials, the capacitance and inductance of the circuits changed, and then it influenced the resonant frequency of metamaterials. Based on this principle, metamaterials may have potential in substance detecting. In order to verify the ability of liquid-metal-based metamaterials to detect different substance molecules, glucose, urea, and sodium chloride were deposited onto fabricated metamaterials separately. During the experiment, the glucose, urea, and sodium chloride solutions all had a concentration of 1% by weight and the solvent was deionized water. A 500 μL solution with a different solute was added to the surface of metamaterials, and the metamaterials were dried until the water evaporated completely. Then the S_21_ parameters of these metamaterials were measured. Taking metamaterials with a DSRR structure as an example, the resonant frequency of the blank group was 7.76 GHz. As shown in Figure 6C, when deionized water was added and then dried out, the S_21_ parameter curve of DSRR-based metamaterials was nearly unchanged. The resonant frequency shifted greatest when glucose was deposited, while sodium chloride caused the smallest change. For ESRR-based metamaterials, a similar conclusion could be drawn, as shown in Figure 6D. Consequently, liquid-metal-based metamaterials show benefits in terms of the development of fast, label-free, and low-cost detection of biomolecules.

## 4. Discussion and Perspective

From the above results, it can be seen that liquid-metal-based electromagnetic metamaterials can provide negative permittivity and permeability under certain wave bands. This is consistent with the electromagnetic properties of existing metamaterials. Compared to traditional rigid ones, liquid-metal-enabled transformable metamaterials have the following features:

Firstly, the fabrication process is convenient and straightforward. Liquid-metal printing can be directly conducted via a laptop printer. Although the PCB technique is quite widespread and reliable, the whole process is relatively complex if compared with liquid-metal printing. The newly emerging liquid-metal printing would significantly help to improve the fast fabrication of SRR circuits and make metamaterials more accessible.

Secondly, transformability and flexibility are outstanding features of liquid-metal-based metamaterials. Liquid metal is the combination of fluidity and metallicity, providing unique physical properties. Traditional PCB circuits are rigid, while liquid-metal circuits can be transferred to elastic substrates, such as PDMS and PVA. Therefore, metamaterials fabricated by liquid metal can tolerate high tensile strength that other soft circuits cannot. Thus, they can well fit the targeted object to enhance the sensitivity. Moreover, being transformable and flexible, the resonant frequency can be changed by elastic deformation, which means that a metamaterial circuit can work under different frequencies. Liquid metal can be deformed by an electrical field, magnetic field, and external force. Thus, metamaterials based on liquid metal are transformable, greatly expanding the working limits of conventional metamaterials. The working frequency of liquid-metal-based metamaterials could be regulated, which can help to develop multifunctional metamaterials.

Finally, this implies that more extensible functions for liquid-metal-enabled transformable metamaterials are possible, owing to the special performance of liquid metal in electricity, magnetism, acoustics, optics, and thermotics. Various functions can in fact be added in the future, not just limited to electromagnetic properties alone.

**Figure 7:**
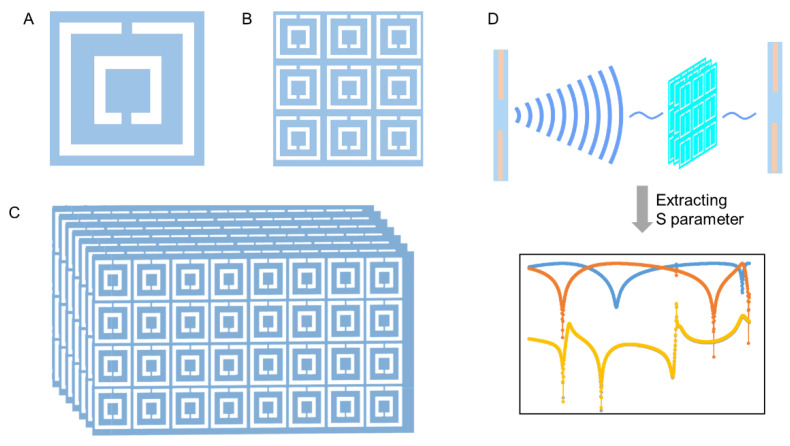
Illustration of metamaterials. (**A**) A single DSRR unit; (**B**) 2D DSRR array; (**C**) 3D DSRR crystal; (**D**) working principle of sensors based on metamaterials.

Due to these significant advantages, the development and applications of liquid-metal metamaterials are promising. Based on fabricated 2D liquid-metal metamaterials, the concept of 3D liquid-metal electromagnetic metamaterials can be further interpreted. As shown in Figure 7A–C, a single DSRR unit can be combined to form plane metamaterials, and then layers of plane DSRR array can further form a 3D structure. Herein, a DSRR unit can be thought of as an atom in crystals. Crystals are constructed through the regular arrangement of atoms in three-dimensional space. Similarly, the regular arrangement of DSRR units in three-dimensional space will form metamaterial crystals. It is clear that more complex structures can be realized on the basis of the DSRR unit, and the dimensions of DSRR units determine the working characteristics of metamaterial crystals.

The potential of liquid-metal metamaterials in sensing has been preliminarily explored, including direction monitoring and substance detecting. In the future, metamaterials are expected to detect more conditions that can cause a shift in resonant frequency. Figure 7D presents the sensing principles of metamaterials based on DSRR structure. Changes in the object being monitored will influence the electromagnetic properties of metamaterials, which can be reflected by the S parameter. By establishing the relationship between the conditions of the object and the S parameter of metamaterials, information can then be obtained through extracting and analyzing the S parameter curve. Compared to traditional biosensors, biosensors based on liquid-metal metamaterials offer a new approach for biomedical detecting. Metamaterial biosensors have the advantages of high sensitivity, high resolution, high miniaturization, high cost-performance, and untagged detection. The combination of liquid metal and metamaterial greatly promotes the development of flexible and multi-functional biosensors.

## 5. Conclusions

In summary, this article investigated the concept of transformable liquid-metal metamaterials and successfully fabricated the typical unit of DSRR and ESRR resonators as practical metamaterials examples. The electromagnetic properties and sensing application of these units have been evaluated, and the feasibility of liquid-metal-based metamaterials has been illustrated. Compared to traditional metamaterials, liquid-metal metamaterials offer quite a few specific advantages owing to their unique characteristics. More research should be conducted in the future to quantify the working properties of liquid-metal-based transformable metamaterials, including their stability, reliability, and repeatability. Meanwhile, fabrication technology based on liquid metal deserves more attention. It is promising to combine liquid metal and metamaterials together to realize more interesting functions in the near future.

## 6. Materials and Method

*Liquid metal.* The liquid metal used in this research was a gallium–indium alloy (EGaIn), containing 75.5 wt% gallium and 24.5 wt% indium. To achieve this alloy, gallium was heated by water bath in a beaker for a few minutes. Then, some indium was added according to the above mix ratio, and the mixture of gallium and indium was stirred constantly with a magnetic agitator under 80 °C for more than one hour to make the alloy fully interfused and well distributed. Finally, micro nickel particles were added and mixed up with EGaIn to form Ni-EGaIn, for the sake of enhancing the effect of selective adhesion [49].

*Printing approach.* As the first trial, we employed a recently proposed printing method that is convenient for laboratory use but has not been standardized and industrialized [50]. The printing substrate used here was thermal transfer paper, covered by a layer of polyurethane glue. Ni-EGaIn shows strong adhesion to polyurethane but hardly adheres to toners. Then, toners were printed on the thermal transfer paper by a laser printer to cover the surface to which we did not want Ni-EGaIn to adhere. Afterwards, a roller was applied to pattern Ni-EGaIn, and the designed circuit was finally formed, which can be transferred to diverse elastic substrates by imprinting. Figure 3A shows the detailed process of SRR unit fabrication.

*Simulation.* The electromagnetic simulation software CST Microwave Studio was adopted to simulate the designed structure.

*Experimental evaluating.* To evaluate the electromagnetic properties of the liquid-metal metamaterials based on SRR arrays, we used a microwave analyzer (Keysight N9951A) and a waveguide to test the S parameters. 

## Data Availability

The data presented in this study are available on request to the correspondent author’s email with appropriate justification.
